# Supplementary material for: Effects of Celastrol-Enriched Peanuts on Metabolic Health and the Development of Atherosclerosis
Source: Nutrients. 2025 Apr 23;17(9):1418. doi: 10.3390/nu17091418 (PMC12073367; doi:10.3390/nu17091418)
Supplement: Supplementary file 1 [file nutrients-17-01418-s001.zip › nutrients-3561398-supplementary.pdf]

Supplement materials:

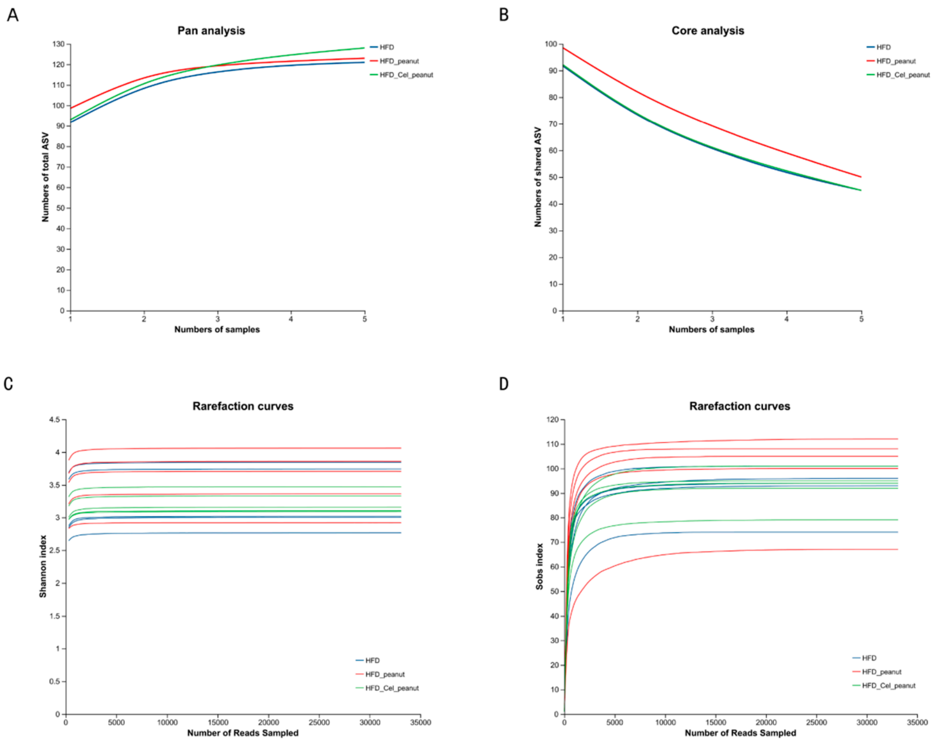

Figure S1. Microbial species analysis and dilution curve. (**A**) Intestinal flora Pan species analysis; (**B**) Intestinal flora Core species analysis; (**C**) Intestinal flora dilution curve (Shannon index); (**D**) Intestinal flora dilution curve (Sobs index).

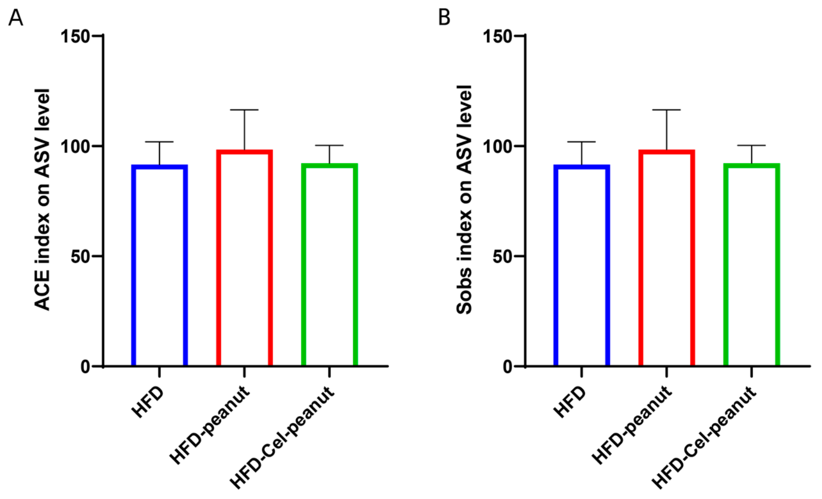

Figure S2. Effect of Cel-peanut intervention on the richness of intestinal flora. (**A**) Intestinal flora alpha-diversity (ACE index); (**B**) Intestinal flora alpha-diversity (Sobs index). Data results are expressed in the form: mean ± standard error, n = 5, indicating that there are 5 mice.

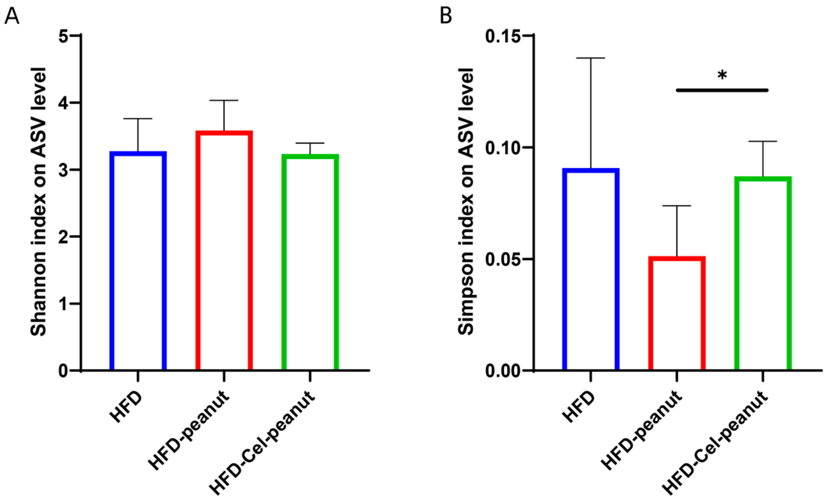

Figure S3. Effect of Cel-peanut intervention on intestinal flora diversity. (**A**) Intestinal flora alpha-diversity (Shannon index); (**B**) Intestinal flora alpha-diversity (Simpson index). Data results are expressed in the form: mean ± standard error, n = 5, indicating that there are 5 mice. * $p < 0.05$, with significant differences.

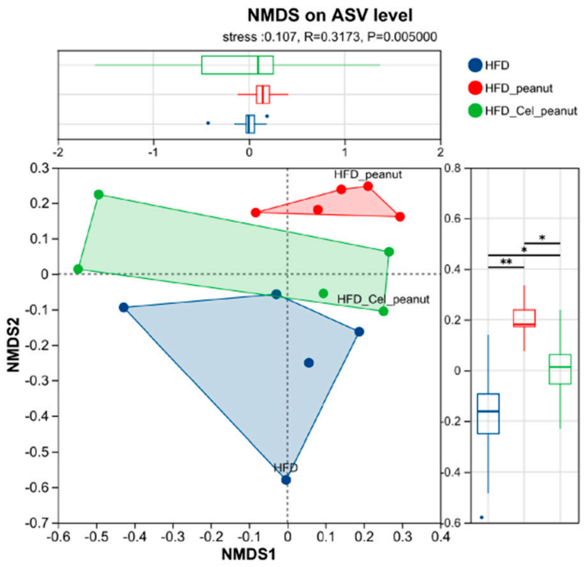

Figure S4. NMDS analysis of intestinal microbiota and box plot based on PC1 and PC2 axis. * $p <$ 0.05, ** $p < 0.01$ with significant differences.

Table S1. Sample name and information.

Sample	Series Number	Bases Number（bp）	Average Length（bp）	Minimum Length（bp）	Maximum Length（bp）
HFD_1	56919	23954379	420.850314	236	446
HFD_2	67291	28111307	417.757308	336	446
HFD_3	66348	28199133	425.018584	283	502
HFD_4	70174	29629323	422.226508	201	437
HFD_5	64764	26819523	414.11159	215	431

HFD_peanut_1	75449	31322103	415.142719	262	432
HFD_peanut_2	73678	30673809	416.322498	261	432
HFD_peanut_3	74809	31568588	421.989172	283	504
HFD_peanut_4	82443	34421245	417.515677	231	492
HFD_peanut_5	93913	39117052	416.524358	251	432
HFD_Cel_peanut_1	68854	28906384	419.821419	217	534
HFD_Cel_peanut_2	68038	28477601	418.554352	386	433
HFD_Cel_peanut_3	74671	31237466	418.334641	258	467
HFD_Cel_peanut_4	77625	32581008	419.72313	338	443
HFD_Cel_peanut_5	61057	25601916	419.311725	325	466
